# An Expedient Total Synthesis of Triciribine

**DOI:** 10.3390/molecules22040643

**Published:** 2017-04-17

**Authors:** Chen Hu, Zhizhong Ruan, Haixin Ding, Yirong Zhou, Qiang Xiao

**Affiliations:** Jiangxi Key Laboratory of Organic Chemistry, Jiangxi Science & Technology Normal University, Nanchang 330013, China; nchuchen@yahoo.com (C.H.); ruanzhizhong87@163.com (Z.R.); dinghaixin_2010@163.com (H.D.); zyrhust@126.com (Y.Z.)

**Keywords:** triciribine, ring closure, nucleosides, glycosylation, total synthesis

## Abstract

In the present paper, we report an expedient total synthesis of triciribine, a tricyclic 7-deazapurine nucleoside and protein kinase B (AKT ) inhibitor, in 35% overall yield. Our synthesis route features a highly regioselective substitution of 1-*N*-Boc-2-methylhydrazine and a trifluoroacetic acid catalyzed one-pot transformation which combined the deprotection of the *tert*-butylcarbonyl (Boc) group and ring closure reaction together to give a tricyclic nucleobase motif.

## 1. Introduction

**Figure 1:**
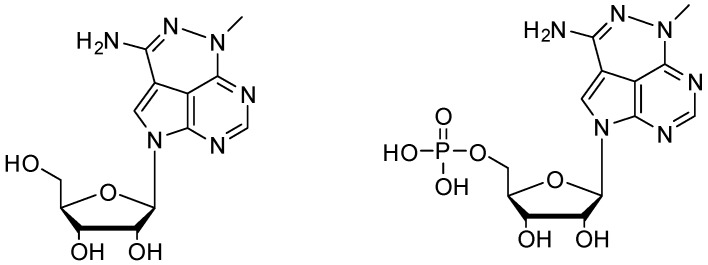
Structures of triciribine **1** and triciribine monophosphate **2**.

In the past few decades, a large number of novel nucleosides have been successfully developed as antiviral and antitumor drugs [1,2]. Triciribine (TCN, **1**, Figure 1), a unique tricyclic 7-deazapurine nucleoside, was firstly synthesized by Townsend in 1971 [3]. Initial biological evaluation of TCN showed excellent activity against murine leukemia cell line L1210 [4]. In order to improve the bioavailability, TCN-P (triciribine phosphate monohydrate, **2**, Figure 1) was further developed to enhance its solubility in water. In the late 1980s, TCN-P was thoroughly studied as a cytotoxic agent in a phase I clinical trial for cancer treatment [5,6]. Unfortunately, it was precluded during the phase II clinical trial in the early 1990s because of its poor toxicity profile and mild efficacy [7,8].

In recent years, TCN-P was reinvestigated as an efficient inhibitor of the protein kinase B (AKT) signaling pathway [9]. It was found that AKT would be over activated in a high percentage of leukemia tumors and a wide range of other solid tumors, including breast, ovarian and pancreatic cancers, etc. [10]. Currently, it is undergoing a phase I/IIa clinical trial for the treatment of breast cancer and pancreatic cancer [11].

**Scheme 1:**
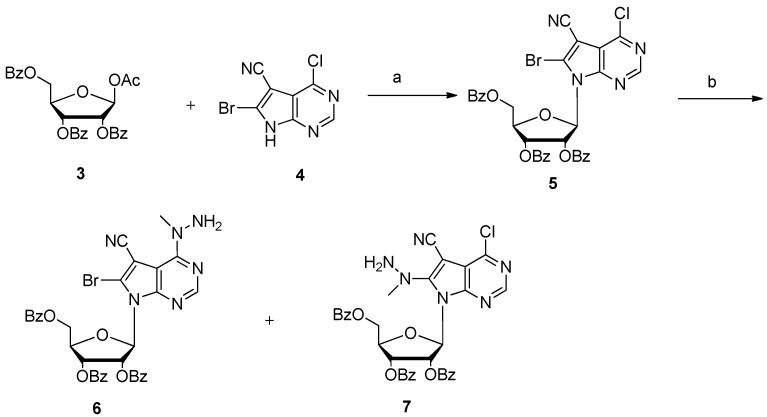
Synthesis of triciribine intermediate. *Reagents and conditions:* (**a**) Method A: *N*,*O*-Bis(trimethylsilyl)acetamide (BSA), CH_3_CN, trimethylsilyl trifluoromethanesulfonate( TMSOTf), 80 °C, 70%; Method B: BSA, CH_3_CN, TMSOTf, microwave heating, 80 °C, 68%; Method C: BSA, CH_3_CN, nonafluorobutane sulfonic acid potassium, TMSCl, microwave heating, 80 °C, 63%; (**b**) NH_2_NHCH_3_, CHCl_3_, EtOH, r.t., 60% (**6**), 16% (**7**).

During our development of a new triciribine prodrug for discovering a potential enhanced AKT inhibitor, ten grams’ scale of triciribine was necessary as a starting material. Therefore, an expedient synthetic procedure was in high demand. However, to the best of our knowledge, there were only three publications concerning its preparation. In the original paper by Townsend [2], the naturally occurring nucleoside toyocamycin was used as starting material, which is extremely expensive. Thirty years later, they reported an improved synthetic approach in 2004 [12]. 6-Bromo-4-chloro-5-cyano-pyrrolo[2,3-*d*]pyrimidine **4** was employed as the nucleoside base to afford nucleoside **5** in high yield and stereoselectivity under standard Vorbrüggen glycosylation condition. Further reaction with methylhydrazine gave nucleoside **6** with exclusive regioselectivity (Scheme 1). After debromination with 10% palladium on charcoal, triciribine was accomplished in moderate yield by refluxing with sodium methoxide in methanol. Later, TSRL Inc. reported another expedient total synthesis approach in 2011 [13]. The key step was the selective introduction of cyanation at 7-position of the deazapurine using tributyltin cyanide and pallidum tetraphenylphosphine. Because nucleobase 4-chloro-5-iodo-*7H*-pyrrolo[2,3-*d*]pyrimidine is commercially available, it could easily scale up triciribine’s preparation. The drawback is that transition-metal-catalyzed cyanation will cause heavy metal residue, which is cumbersome to remove completely [14]. In the present paper, a new expedient total synthesis of triciribine was developed.

## 2. Results and Discussion

In our foregoing synthesis of 7-deazpurine nucleoside [15,16,17], our group gained rich experiences in the process of preparing 6-bromo-4-chloro-5-cyano-pyrrolo[2,3-*d*]pyrimidine **4** in 100 g scale. At the beginning, we tried to synthesize triciribine by Townsend’s approach (Scheme 1). Thus, nucleobase **4** was firstly silylated with *N*,*O*-bis(trimethylsilyl) acetamide (BSA) in anhydrous CH_3_CN. Then 1-*O*-acetyl-2,3,5-tri-*O*-benzoyl-β-d-ribofuranose **3** was added to the above homogeneous solution, followed by the slow addition of trimethylsilyl trifluoromethanesulfonate (TMSOTf). Subsequently, the reaction mixture was heated to 80 °C for 10 h, and 7-deazapurine nucleoside **5** was obtained successfully with 70% yield. During our extensive optimization of glycosylation, it was found that the combination of nonafluorobutane sulfonic acid potassium and trimethylsilyl chloride was superior to TMSOTf and that microwave heating can speed up the reaction. With sufficient nucleoside **5** in hand, it was then reacted with methylhydrazine in ethanol. To our surprise, besides the desired nucleoside **6** with 60% yield, another byproduct **7** was also separated in 16% yield. The structure of nucleoside **7** was established by high resolution mass spectroscopy (HRMS ) and various NMR spectra. Because of the electron withdrawing properties of the cyano group, the neibouring 8-bromo group could be easily substituted by the nucleophilic reagent. This phenomenon was also found in other 7-deazapurine nucleosides [18,19,20]. To depress the side reaction, various methylhydrazine from different commercial sources and a variety of solvents were investigated. However, the results showed that it seemed impossible to exclude the side reaction completely.

**Scheme 2:**
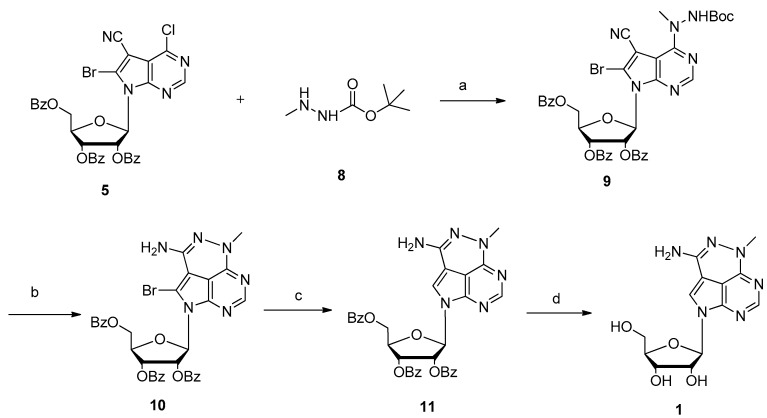
Total synthesis of triciribine **1**. *Reagents and conditions:* (**a**) CH_3_NHNHBoc, CH_2_Cl_2_, EtOH, triethylamine (TEA), reflux, 89%; (**b**) trifluoroacetic acid (TFA), CH_2_Cl_2_, r.t., 74%; (**c**) 10% Palladium on carbon (Pd/C), TEA, THF, CH_3_OH, H_2_, r.t., 83%; (**d**) CH_3_ONa, CH_2_Cl_2_, CH_3_OH, r.t., 92%.

**Figure 2:**
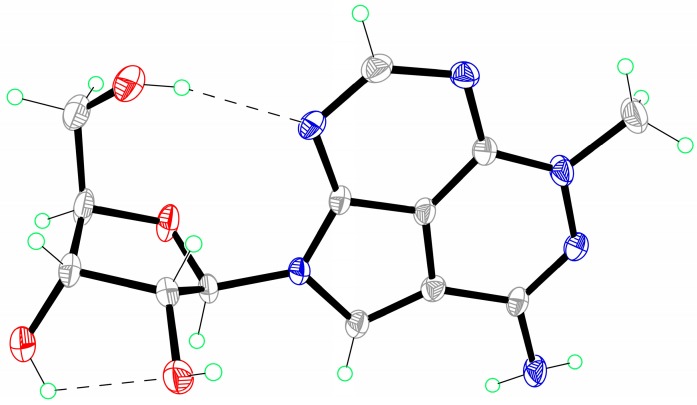
X-ray crystal structure of triciribine.

To improve the total synthetic yield of triciribine, we reasoned that a practicable way could be by decreasing the nucleophilicity of methylhydrazine to alleviate the side reaction (Scheme 2). Commercially available 1-*N*-Boc-2-methylhydrazine has an electron-withdrawing *tert*-butylcarbonyl (Boc) group at N-1 position, which could reduce the NH-2 nucleophilicity of methylhydrazine [21,22,23,24]. To our delight, nucleoside **9** could be obtained in 89% after treating nucleoside **5** with 1-Boc-2-methylhydrazine by using triethylamine as a base in a mixture solvent of anhydrous dichloromethane (DCM) and ethanol. The 8-bromo substituted side product was not detected. Then, trifluoroacetic acid (TFA) was utilized to remove the Boc group. It was surprising to find that the tricyclic nucleoside **10** was generated directly by the TFA promoted ring closure reaction in situ. In the previously reported literature [12,13], the tricyclic nucleobase motif was achieved by heating nucleoside **6** with sodium methoxide in methanol at reflux temperature for 18 h. Then, debromination was accomplished with 10% Pd/C in ethanol under refluxing for 5 h to afford nucleoside **11** with 83% yield. At last, treatment **11** with sodium methoxide in methanol at room temperature provided triciribine **1** in 92% yield. For the first time, a suitable single crystal of triciribine **1** was obtained from methanol and H_2_O (10:1) and its structure is unambiguously confirmed by X-ray diffraction analysis (Figure 2) [25]. It exhibits a *syn* glycosylic bond conformation, which is stabilized by an intramolecular O–H···N hydrogen bond.

## 3. Materials and Methods

All the chemicals and compounds were used as received except for special notification. ^1^H NMR spectra and ^13^C NMR spectra were recorded on a Bruker Advanced DPX spectrometer (400 MHz for ^1^H NMR and 100 MHz for ^13^C NMR, Bruker Corporation, Billerica, MA, USA). Chemical shifts are reported in ppm from tetramethylsilane with the solvent resonance as the internal standard. Data are reported as follows: chemical shift, multiplicity (s = singlet, d = doublet, t = triplet, q = quartet, m = multiplet, br = broad), coupling constants (Hz) and integration. ^13^C NMR spectra were recorded with complete proton decoupling. HRMS was measured on AB Sciex 4600 mass spectrometer equipped with an ESI source in the positive-ion mode (Bruker Corporation, Billerica, MA, USA). Melting points were measured on an X-4 digital melting point apparatus (Beijing Taike Corparation, Beijing, China). The microwave synthesizer was equipped with Anton Paar autosampler MAS24 Monowave 300 (Anton Paar GmbH, Graz, Austria). X-ray analysis was recorded on Bruker Smart Apex II (Bruker Corporation, Billerica, MA, USA). Specific rotation was recorded on Rudolph Autopol IV (Rudolph Research Analytical, Hackettstown, NJ, USA).

### 3.1. Synthesis of 6-Bromo-4-chloro-5-cyano-7-[2,3,5-tri-O-benzoyl-β-d-ribofuranosyl]pyrrolo[2,3-d]pyrimidine *(**5**)*

**Method A**: 6-Bromo-4-chloro-5-cyanopyrrolo[2,3-*d*]pyrimidine **4** (257 mg, 1 mmol) was suspended in freshly distilled dry acetonitrile (15 mL) and then *N*,*O*-bis(trimethylsilyl)acetamide (BSA, 2.44 g, 12 mmol) was added to the reaction under an argon atmosphere. The reaction mixture was stirred at room temperature for 10 min. Then 1-*O*-acetyl-2,3,5-tri-*O*-benzoyl-β-d-ribofuranose **3** (770 mg, 1.5 mmol) was added along with trimethylsilyl trifluoromethanesulfonate (TMSOTf, 890 mg, 4 mmol). After stirring for 15 min, the mixture was heated at 80 °C for 4 h until Thin layer chromatography (TLC) analysis showed that the starting material was consumed completely. Then it was cooled to room temperature, quenched with iced water (20 mL) and extracted with ethyl acetate (20 mL × 3). The combined organic layer was washed with saturated sodium bicarbonate aqueous solution (30 mL) and brine (30 mL), respectively, and dried over magnesium sulfate. After filtration, the solvent was removed under vacuum. The crude product was dissolved in chloroform and purified by silica gel column chromatography to provide the product **5** as a white solid 490 mg, 70% yield.

**Method B**: 6-Bromo-4-chloro-5-cyanopyrrolo[2,3-*d*]pyrimidine **4** (257 mg, 1 mmol) was suspended in freshly distilled dry acetonitrile (15 mL) and then *N*,*O*-bis(trimethylsilyl)acetamide (2.44 g, 12 mmol) was added to the reaction under an argon atmosphere. The reaction mixture was stirred at room temperature for 10 min. Then 1-*O*-acetyl-2,3,5-tri-*O*-benzoyl-β-d-ribofuranose **3** (770 mg, 1.5 mmol) was added along with trimethylsilyl trifluoromethanesulfonate (TMSOTf, 890 mg, 4 mmol). Then the reaction mixture was transferred to a microwave synthesizer, and heated at 80 °C for 50 min until TLC analysis showed that the starting material was consumed completely. Then it was cooled to room temperature, quenched with ice water (20 mL) and extracted with ethyl acetate (15 mL × 3). The combined organic layer was washed with saturated sodium bicarbonate aqueous solution (30 mL) and brine (30 mL), respectively, and dried over magnesium sulfate. After filtration, the solvent was removed under vacuum. The crude product was dissolved in chloroform and purified by silica gel column chromatography to provide the product **5** as a white solid 477 mg, 68% yield.

**Method C**: *N*,*O*-Bis(trimethylsilyl)acetamide (1.38 g, 6.8 mmol), 6-bromo-4-chloro-5-cyanopyrrolo[2,3-*d*]pyrimidine **4** (460 mg, 1.95 mmol), 1-*O*-acetyl-2,3,5-tri-*O*-benzoyl-β-d-ribofuranose **3** (1.55 g, 3.1 mmol), nonafluorobutane sulfonic acid potassium (3.2 g, 9.4 mmol) and trimethylsilyl chloride (TMSCl, 1.02 g, 9.4 mmol) were added in freshly distilled dry acetonitrile (15 mL). Then the reaction mixture was transferred to a microwave synthesizer, and heated at 80 °C for 50 min until TLC analysis showed that the starting material was consumed completely. Then it was cooled to room temperature, quenched with ice water (20 mL) and extracted with ethyl acetate (20 mL × 3). The combined organic layer was washed with saturated sodium bicarbonate aqueous solution (30 mL) and brine (30 mL), respectively, and dried over magnesium sulfate. After filtration, the solvent was removed under vacuum. The crude product was dissolved in chloroform and purified by silica gel column chromatography to provide the product **5** as a white solid 800 mg, 63% yield.

Retention factor (R*_f_*) = 0.4 (CH_2_Cl_2_/EtOAc, 40:1); m.p. 88–91 °C (Lit.^9^ 100 °C); ${\left[\alpha \right]}_{D}^{25}$ −32.184 (*c* = 0.087 g/100 mL, EtOAc); ^1^H NMR (400 MHz, DMSO-*d*_6_) δ: 8.70 (s, 1H, H-2), 7.95–7.83 (m, 6H), 7.67–7.64 (m, 3H), 7.50–7.40 (m, 6H), 6.59 (d, *J* = 3.2 Hz, 1H, H-1′), 6.55 (dd, *J* = 6.0, 3.2 Hz, 1H, H-2′), 6.41 (t, *J* = 6.8 Hz, 1H, H-3′), 4.93 (dt, *J* = 7.2, 3.6 Hz, 1H, H-4′), 4.84 (dd, *J* = 12.4, 2.8 Hz, 1H, H-5′), 4.60 (dd, *J* = 12.4, 4.2 Hz, 1H, H-5′). ^13^C NMR (100 MHz, DMSO-*d*_6_) δ: 165.7, 165.1, 152.9 (C-6), 150.8 (C-2), 150.7 (C-4), 134.5, 134.4, 133.9, 129.9, 129.8, 129.5, 129.3, 129.2, 129.1, 128.9, 128.8, 116.9 (C-8), 113.2 (CN), 101.8 (C-5), 89.8 (C-1′), 89.5 (C-7), 79.6 (C-4′), 73.6 (C-2′), 70.3 (C-3′), 62.8 (C-5′); MS (ESI) *m*/*z*: 702.9 [M + H]^+^. HR-MS: calcd. for C_33_H_22_BrClN_4_O_7_ [M + Na]^+^ 725.0235; found: 725.0239.

### 3.2. Synthesis of 6-Bromo-5-cyano-4-(1-methylhydrazino)-7-[2,3,5-tri-O-benzoyl-β-d-ribofuranosyl]pyrrolo[2,3-d]pyrimidine *(**6**)*

6-Bromo-4-chloro-5-cyano-7-[(2,3,5-*O*-tribenzoyl-yl)-β-d-ribofuranosyl]pyrrolo[2,3-*d*]pyrimidine **5** (0.47 g, 0.67 mmol) was dissolved in a mixture of chloroform (7.5 mL) and ethanol (7.5 mL), then the reaction mixture was treated with the solution of methyl hydrazine (40%, 0.24 g, 2.08 mmol). The reaction was stirred at room temperature for 3 h while a white precipitate was formed. TLC analysis showed that the starting material was consumed completely. The solvent was removed under vacuum and the crude product was purified by silica gel column chromatography to provide the product **5** as a white solid. 286 mg, 60% yield; R*_f_* = 0.5 (CH_2_Cl_2_/EtOAc, 30:1); m.p. 192–195 °C; ${\left[\alpha \right]}_{D}^{25}$ −45.763 (*c* = 0.118 g/100 mL, CH_2_Cl_2_); ^1^H NMR (400 MHz, DMSO-d_6_) δ: 8.13 (s, 1H, H-2), 7.91 (t, *J* = 6.4 Hz, 6H), 7.65 (t, *J* = 7.2 Hz, 3H), 7.54–7.37 (m, 6H), 6.58 (d, *J* = 4.0 Hz, 1H, H-1′), 6.43 (d, *J* = 8.0 Hz, 1H, H-2′), 6.39 (d, *J* = 8.0 Hz, 1H, H-3′), 5.25 (s, 2H, NH_2_), 4.91-4.83 (m, 1H, H-4′), 4.78 (d, *J* = 12.2, 1H, H-5′), 4.60 (dd, *J* = 12.0, 4.0 Hz, 1H, H-5′), 3.35 (s, 3H, CH_3_). ^13^C NMR (100 MHz, DMSO-*d*_6_) δ: 165.8, 165.2, 157.2 (C-6), 152.8 (C-2), 151.4 (C-4), 134.5, 134.3, 134.0, 129.9, 129.8, 129.7, 129.3, 129.2, 129.1, 129.0, 128.9, 121.9, 116.6 (CN), 101.5 (C-5), 94.2 (C-1′), 89.7, 79.1 (C-4′), 73.5 (C-2′), 70.5 (C-3′), 63.0 (C-5′), 40.6 (CH_3_). MS (ESI) *m*/*z*: 711.1 [M + H]^+^. HR-MS: calcd. for C_34_H_27_BrN_6_O_7_ [M + Na]^+^ 733.1017; found: 733.1028.

Besides the desired product **6**, a small amount of by-product **7** was isolated. 22 mg 16% yield. R*_f_* = 0.53 (CH_2_Cl_2_/ EtOAc, 30:1); m.p. 189–192 °C; ${\left[\alpha \right]}_{D}^{25}$ −57.843 (*c* = 0.102 g/100 mL, CH_2_Cl_2_); ^1^H NMR (400 MHz, DMSO-*d*_6_) δ: 8.41 (s, 1H, H-2), 7.90 (dd, *J* = 14.8, 7.4 Hz, 6H), 7.68–7.62 (m, 3H), 7.51–7.40 (m, 6H), 6.99 (d, *J* = 3.4 Hz, 1H, H-1′), 6.56-6.54 (m, 1H, H-2′), 6.35-6.32 (m, 1H, H-3′), 5.37 (s, 2H, NH_2_), 4.84–4.76 (m, 1H, H-4′), 4.74 (dd, *J* = 6.8, 4.0 Hz, 1H, H-5′), 4.61 (dd, *J* = 12.0, 4.4 Hz, 1H, H-5′), 3.42 (s, 3H, CH_3_); ^13^C NMR (100 MHz, DMSO-*d*_6_) δ: 165.8, 165.1, 165.0, 157.3, 150.7 (C-2), 150.5, 147.2, 134.33, 134.29, 134.0, 129.84, 129.77, 129.71, 129.67, 129.21, 129.18, 129.15, 129.1, 129.0, 117.2, 115.5 (CN), 88.4 (C-1′), 78.8, 73.2 (C-4′), 70.5 (C-2′), 66.5 (C-3′), 63.2 (C-5′), 45.5 (CH_3_). HR-MS: calcd. for C_34_H_27_ClN_6_O_7_ [M + Na]^+^ 689.1522; found: 689.1532.

### 3.3. Synthesis of 6-Bromo-5-cyano-4-(2-(tert-butoxycarbonyl)-1-methylhydrazinyl)-7-[2,3,5-tri-O-benzoyl-β-d-ribofuranosyl]pyrrolo[2,3-d]pyrimidine *(**9**)*

6-Bromo-4-chloro-5-cyano-7-[2,3,5-tri-*O*-benzoyl-β-d-ribofuranosyl]pyrrolo[2,3-*d*]pyrimidine **5** (1.75 g, 2.5 mmol), 1-Boc-2-methylhydrazine (0.44 g, 3 mmol) and triethylamine (TEA, 0.3 g, 3 mmol) were added in the mixture of CH_2_Cl_2_ (25 mL) and anhydrous C_2_H_5_OH (25 mL). The reaction mixture was refluxed under an argon atmosphere for 72 h. TLC analysis showed that the starting material was consumed and a new spot appeared. The mixture was poured over saturated sodium bicarbonate solution (40 mL × 2) and extracted with dichloromethane (50 mL × 3), the organic layer was then washed with brine (30 mL × 2) and dried over magnesium sulfate. After filtration, the solvent was removed under vacuum. The crude product was dissolved in dichloromethane and purified by silica gel column chromatography to provide the product **9** as a white solid. 1.82 g, 89% yield; R*_f_* = 0.4 (CH_2_Cl_2_/EtOAc, 80:1); m.p. 120–123 °C; ${\left[\alpha \right]}_{D}^{25}$ −41.379 (*c* = 0.087 g/100 mL, CH_3_OH); ^1^H NMR (400 MHz, DMSO-*d*_6_) δ: 9.68 (s, 1H, NH), 8.29 (s, 1H, H-2), 7.91 (d, *J* = 6.0 Hz, 6H), 7.67–7.63 (m, 3H), 7.49–7.43 (m, 6H), 6.55 (s, 1H, H-2′), 6.48 (s, 1H, H-1′), 6.42 (s, 1H, H-3′), 4.89–4.87 (m, 1H, H-4′), 4.78 (d, *J* = 12.2 Hz, 1H, H-5′), 4.61 (d, *J* = 8.8 Hz, 1H, H-5′), 3.29 (s, 3H, 6-N-CH_3_), 1.44 (s, 9H, Boc). ^13^C NMR (100 MHz, DMSO-*d*_6_) δ: 165.5, 164.9, 164.8, 156.9, 155.2, 152.4, 151.2 (C-2), 134.1, 134.0, 133.6, 129.52, 129.48, 129.3, 128.91, 128.86, 128.81, 128.7, 128.6, 124.0, 114.9 (CN), 101.1, 90.9, 89.6 (C-1′), 81.0 (Boc), 78.9 (C-4′), 73.2 (C-2′), 70.1 (C-3′), 62.7 (C-5′), 37.9 (CH_3_), 28.0 (Boc). MS (ESI) *m*/*z*: 811.0 [M + H]^+^. HR-MS: calcd. for C_39_H_35_BrN_6_O_9_ [M + Na]^+^ 833.1541; found: 833.1552.

### 3.4. Synthesis of 6-Amino-7-bromo-4-methyl-8-(2,3,5-tri-O-benzoyl-β-d-ribofuranosyl)pyrrolo[4,3,2-de]pyrimido [4,5-c]pyridazine *(**10**)*

Nucleside **9** (1.73 g, 2.1 mmol) was added in the mixture of trifluoroacetic acid (TFA, 20 mL) and dichloromethane (20 mL). The reaction mixture was stirred at room temperature for 2 h. TLC showed that the starting material was consumed and a new spot appeared. The mixture was poured over water (100 mL) and extracted with dichloromethane (50 mL × 2). The organic layer was neutralized with triethylamine and dried over magnesium sulfate. After filtration, the solvent was removed under vacuum. The crude product was dissolved in dichloromethane and purified by silica gel column chromatography to provide the product **10** as a white solid. 1.3 g, 74% yield; R*_f_* = 0.4 (CH_2_Cl_2_/EtOAc, 40:1); m.p. 102–105 °C; ${\left[\alpha \right]}_{D}^{25}$ −34.426 (*c* = 0.122 g/100 mL, CH_3_OH); ^1^H NMR (400 MHz, DMSO-*d*_6_) δ: 8.03 (s, 1H, H-7), 7.94–7.88 (m, 6H), 7.67–7.63 (m, 3H), 7.48–7.41 (m, 6H), 6.79 (t, *J* = 3.6 Hz, 1H, H-1′), 6.39 (t, *J* = 6.2 Hz, 1H, H-2′), 6.33 (d, *J* = 3.4 Hz, 1H, H-3′), 5.87 (s, 2H, NH_2_), 4.85 (d, *J* = 2.8 Hz, 1H, H-4′), 4.77 (d, *J* = 12.0 Hz, 1H, H-5′), 4.56 (dd, *J* = 12.2, 3.4 Hz, 1H, H-5′), 3.38 (s, 3H, CH_3_). ^13^C NMR (100 MHz, DMSO-*d*_6_) δ: 165.8, 165.1, 165.0, 157.0, 150.7, 147.4, 144.6 (C-3), 134.4, 134.3, 133.8, 129.83, 129.76, 129.6, 129.2, 129.1, 129.0, 128.8, 109.3, 105.1, 93.2 (C-2), 88.1 (C-1′), 79.3 (C-4′), 72.9 (C-2)′, 70.6 (C-3′), 62.9 (C-5′), 35.8 (CH_3_). MS (ESI) *m*/*z*: 711.0 [M + H]^+^. HR-MS: calcd. for C_34_H_27_BrN_6_O_7_ [M + Na]^+^ 733.1017; found: 733.1014.

### 3.5. Synthesis of 6-Amino-4-methyl-8-(2,3,5-tri-O-benzoyl-β-d-ribofuranosyl)pyrrolo[4,3,2-de]pyrimido[4,5-c]pyridazine *(**11**)*

Nucleoside **10** (1.2 g, 1.45 mmol), triethylamine (0.734 g, 7.3 mmol), and 10% palladium on activated charcoal (180 mg) were added in a mixture of tetrahydrofuran (THF) (20 mL) and methanol (10 mL). The reaction mixture was stirred at room temperature under H_2_ atmosphere (1 atm) for 3 h. TLC analysis showed that the starting material was consumed completely. The reaction mixture was filtered through Celite and washed with hot ethanol (50 mL). The solvent was removed under vacuum and the crude product was purified by silica gel column chromatography to provide the product **11** as a white solid. 0.9 g, 83% yield; R*_f_* = 0.2 (CH_2_Cl_2_/ EtOAc, 1:1); m.p. 104–107 °C; ${\left[\alpha \right]}_{D}^{25}$ −48.598 (*c* = 0.107 g/100 mL, CH_3_OH); ^1^H NMR (400 MHz, DMSO-*d*_6_) δ 8.02 (s, 1H, H-7), 8.01–7.86 (m, 6H), 7.70–7.59 (m, 3H), 7.46–7.44 (m, 6H), 7.16 (s, 1H, H-2), 6.55 (d, *J* = 4.2 Hz, 1H, H-1′), 6.48 (t, *J* = 5.2 Hz, 1H, H-2′), 6.30 (s, 2H, NH_2_), 6.21 (t, *J* = 5.8 Hz, 1H, H-3′), 4.90–4.80 (m, 1H, H-4′), 4.76 (dd, *J* = 12.2, 2.8 Hz, 1H, H-5′), 4.64 (dd, *J* = 12.2, 4.2 Hz, 1H, H-5′), 3.40 (s, 3H, CH_3_). ^13^C NMR (100 MHz, DMSO-*d*_6_) δ 165.7, 164.9, 164.8, 156.5, 151.2, 146.6, 145.8, 134.2, 134.1, 133.7, 129.61, 129.58, 129.53, 129.45, 129.0, 128.96, 128.9, 128.8, 128.6, 111.0, 109.2, 104.3, 88.2 (C-1′), 79.2 (C-4′), 73.4 (C-2′), 70.9 (C-3′), 63.5 (C-5′), 35.5 (CH_3_). MS (ESI) *m*/*z*: 633.2 [M + H]^+^. HR-MS: calcd. for C_34_H_28_N_6_O_7_ [M + Na]^+^ 655.1912; found: 655.1924.

### 3.6. Synthesis of 6-Amino-4-methyl-8-(β-d-ribofuranosyl)pyrrolo[4,3,2-de]pyrimido[4,5-c]pyridazine (Triciribine, TCN, ***1***)

Nucleoside **11** (1.5 g, 2.0 mmol) and sodium methoxide (0.65 g, 12 mmol) were added in a mixture of CH_2_Cl_2_ (5 mL) and CH_3_OH (20 mL). The reaction mixture was stirred at room temperature for 2 h until TLC analysis showed that the starting material was consumed completely. The solvent was removed under vacuum and the yellow residue was suspended in ethyl acetate (10 mL), and neutralized with glacial acetic acid. The residue was filtered and washed with deionized water (10 mL × 3), and dried under vacuum at 80 °C to provide the target product TCN, **1** as a white solid. 0.60 g, 92% yield; R*_f_* = 0.3 (CH_2_Cl_2_/CH_3_OH, 10:1); m.p. 149–150 °C; ${\left[\alpha \right]}_{D}^{25}$ −70.248 (*c* = 0.121 g/100 mL, CH_3_OH); ^1^H NMR (400 MHz, DMSO-*d*_6_) δ: 8.03 (s, 1H, H-7), 7.07 (s, 1H, H-2), 6.28 (s, 2H, NH_2_), 5.81 (d, *J* = 8.0 Hz, 1H, H-1′), 5.63 (q, *J* = 4.0 Hz, 1H, H-2′), 5.41 (d, *J* = 4.0 Hz, 1H, H-3′), 5.22 (d, *J* = 4.0 Hz, 1H, H-4′), 4.51~4.47 (dd, *J* = 12.0, 8.0 Hz, 1H, H-5′), 4.10 (q, *J* = 4.0 Hz, 1H, H-5′), 3.98~3.96 (m, 1H, OH), 3.67–3.62 (m, 1H, OH), 3.59~3.52 (m, 1H, OH), 3.39 (s, 3H, CH_3_). ^13^C NMR (100 MHz, DMSO-*d*_6_) δ: 156.3, 151.6, 146.4, 146.3, 110.8, 109.6, 103.8, 90.0 (C-1′), 86.5 (C-4′), 74.4 (C-2′), 71.5 (C-3′), 62.5 (C-5′), 35.7 (CH_3_). HR-MS: calcd. for C_13_H_16_N_6_O_4_ [M + H]^+^ 321.1306; found: 321.1306.

## 4. Conclusions

In summary, an expedient total synthesis of triciribine was accomplished with 35% total yield. Its merits include: (1) 1-*N*-Boc-2-methylhydrazine could selectively substitute 6-chloro group of nucleoside **5** in high yield without any detectable side product; (2) deprotection of the Boc group with TFA could facilitate the ring closure reaction in a one-pot operation to give the tricyclic nucleobase motif; (3) the current newly developed approach has high overall yield and producibility. Developing novel prodrugs of triciribine to discover a potential enhanced AKT inhibitor is undergoing in our group.
